# Supplementary material for: Consumption of Low Dose Fucoxanthin Does Not Prevent Hepatic and Adipose Inflammation and Fibrosis in Mouse Models of Diet-Induced Obesity
Source: Nutrients. 2022 May 29;14(11):2280. doi: 10.3390/nu14112280 (PMC9183127; doi:10.3390/nu14112280)
Supplement: Supplementary file 1 [file nutrients-14-02280-s001.zip › nutrients-1656524-supplementary.pdf]

Supplementary Figure S1

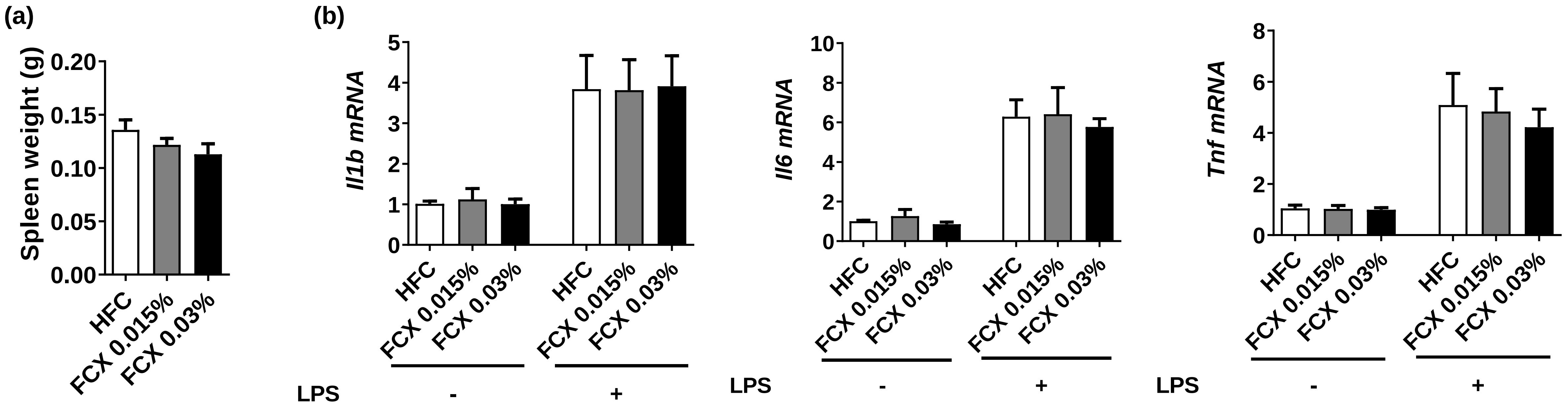

Supplementary Figure S1. Effect of FCX supplementation in splenocytes from a NASH mouse model. Male C57BL/6J mice were fed with either HFC, FCX 0.015%, or FCX 0.03% for 12 weeks. (**a**) Spleen weight. (**b**) mRNA expression of pro-inflammatory cytokines in the splenocytes. Splenocytes were isolated from the mice and stimulated with 500 ng/ml LPS for 20 h to measure *Il1b*, *Il6*, and *Tnf* mRNA expression. n = 6-9 per group. Data shown are mean ± SEM.
